# Targeting Kinase Suppressor of Ras 1 (KSR1) for Cancer Therapy

**DOI:** 10.3390/pharmaceutics17101348

**Published:** 2025-10-19

**Authors:** Hyuk Moon, Hyunjung Park, Soyun Lee, Sangjik Lee, Simon Weonsang Ro

**Affiliations:** Department of Genetics and Biotechnology, College of Life Sciences, Kyung Hee University, Yongin-si 17104, Gyeonggi-do, Republic of Korea; hmoon@khu.ac.kr (H.M.); molly921@khu.ac.kr (H.P.); lsoyun15@khu.ac.kr (S.L.); lsjik0615@gmail.com (S.L.)

**Keywords:** kinase suppressor of Ras 1, RAS/MAPK signaling, targeted therapy, scaffold protein, hepatocellular carcinoma

## Abstract

Carcinogenesis is driven by aberrant activation of molecular signaling pathways governing cell proliferation, apoptosis, and differentiation. Among these, the RAS/RAF/MEK/ERK (RAS/MAPK) cascade is one of the most frequently dysregulated oncogenic pathways, driving tumor initiation and progression across diverse cancer types. Although inhibitors of BRAF and MEK have achieved clinical success in selected malignancies, adaptive resistance often undermines therapeutic durability. This has spurred interest in alternative nodes within the pathway. The kinase suppressor of Ras (KSR) is a scaffold protein that organizes RAF, MEK, and ERK into functional complexes, ensuring efficient and sustained signal transmission. Once regarded as a passive structural component, KSR1 is now recognized as an active regulator of pathway dynamics. Emerging evidence indicates that KSR1 overexpression promotes cancer cell proliferation and survival, while genetic or pharmacologic inhibition of KSR1 attenuates RAS/MAPK signaling and suppresses tumor growth in preclinical models. In this review, we provide a comprehensive overview of accessory and scaffold proteins modulating the RAS/MAPK pathway, with a particular focus on KSR1. We highlight its structural and functional properties, summarize preclinical evidence for KSR1-targeted interventions, and discuss its therapeutic potential in cancer, with emphasis on hepatocellular carcinoma (HCC).

## 1. Introduction

Carcinogenesis is a multistep process driven by the accumulation of genetic and epigenetic alterations leading to a constitutive activation of intracellular molecular signaling networks that control proliferation, survival, and differentiation [1,2,3]. Several core oncogenic signaling pathways are recurrently dysregulated in human malignancies, most notably the RAS/RAF/MEK/ERK (RAS/MAPK) cascade, the PI3K/AKT/mTOR pathway, and the JAK/STAT pathway [4,5,6]. Among these, the RAS/MAPK signaling cascade is one of the most frequently activated across cancer types. In most cases, sustained activation of this pathway arises from activating mutations in members of the RAS gene family (*HRAS*, *KRAS*, *NRAS*) or RAF kinases (*ARAF*, *BRAF*, *CRAF*) [4]. For instance, constitutively activating mutations in KRAS are detected in more than 90% of pancreatic ductal adenocarcinomas [7]. As well, over half of colorectal adenocarcinoma harbor activating mutations in RAS or RAF genes, maintaining constitutive RAS/MAPK pathway activation [4]. In papillary thyroid carcinoma, BRAF mutations are particularly prevalent, occurring in up to 70% of patients, and serve as a major driver of aberrant pathway activation in this malignancy [8].

Owing to its pivotal role in tumor initiation and progression, the RAS/MAPK signaling pathway has been a major focus for targeted therapy development [9,10]. For example, small-molecule inhibitors of BRAF have demonstrated significant clinical benefit, particularly in tumors harboring specific activating mutations. However, treatment efficacy is often limited by the emergence of drug-resistant tumor cells, which compromise the durability of therapeutic responses [4,11,12]. These challenges have stimulated interest in targeting alternative nodes within the pathway, including non-catalytic regulators such as scaffold proteins. In particular, kinase suppressor of Ras 1 (KSR1) has emerged as a key modulator of RAS/MAPK signaling and a novel therapeutic target for cancer with pathway activation.

## 2. The RAS/MAPK Signaling Pathway

**Figure 1:**
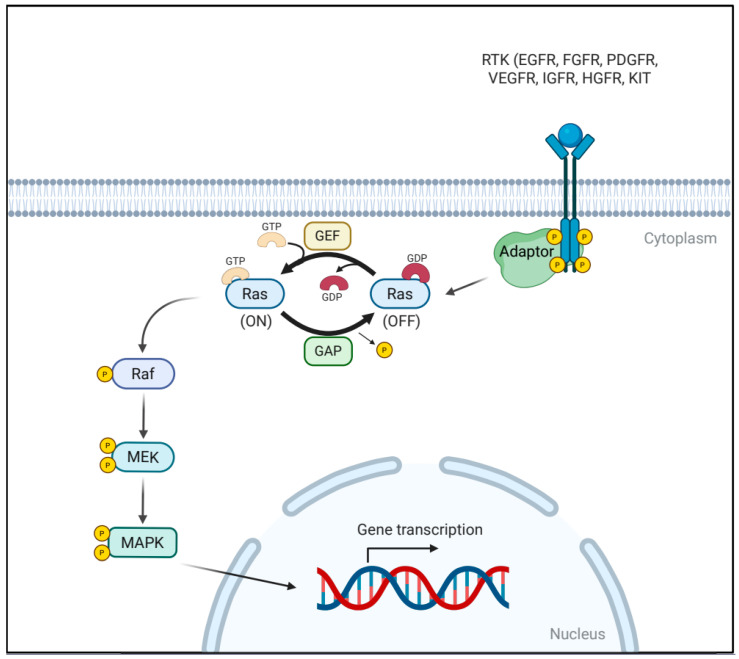
Schematic illustration of RAS/MAPK Signaling Pathway. Ligand-induced receptor dimerization promotes phosphorylation of tyrosine residues within the C-terminal cytoplasmic domain of receptor tyrosine kinases (RTKs). Adaptor proteins bind to these phosphotyrosine sites and recruit guanine nucleotide exchange factors (GEFs), which catalyze the exchange of GDP for GTP on RAS. Activated RAS triggers RAF dimerization, leading to RAF kinase activation and subsequent phosphorylation of MEK. MEK then phosphorylates and activates ERK (MAPK), which translocates into the nucleus to phosphorylate diverse transcription factors, thereby regulating gene expression programs involved in proliferation, survival, and differentiation.

The RAS/MAPK signaling cascade is a highly conserved intracellular signaling pathway that governs fundamental cellular processes, including proliferation, differentiation, survival, and migration. Its activation is typically initiated at the cell surface through ligand binding to transmembrane receptors such as receptor tyrosine kinases (RTKs) and, in some contexts, G-protein-coupled receptors (GPCRs) [13,14]. Upon ligand engagement, RTKs undergo conformational changes that promote dimerization, leading to trans-autophosphorylation of specific tyrosine residues within their cytoplasmic domains (Figure 1). These phosphorylated tyrosines serve as docking sites for adaptor proteins such as growth factor receptor-bound protein 2 (Grb2). Grb2 recruits the guanine nucleotide exchange factor (GEF), son of sevenless (SOS), to the plasma membrane, where it catalyzes the exchange of GDP for GTP on the small GTPase RAS. This molecular switch activates RAS by inducing a conformational change that enables its interaction with downstream effectors, RAF kinases [14,15,16].

Recruited by activated RAS to the plasma membrane, RAF becomes activated through dimerization. Once activated, RAF in turn phosphorylates the mitogen-activated protein kinase kinases, MEK1 and MEK2, on conserved serine residues. MEKs are dual-specificity kinases, capable of phosphorylating both threonine and tyrosine residues, a feature that distinguishes them from most other kinases [17,18]. Activated MEK1/2 phosphorylates extracellular signal-regulated kinases ERK1 and ERK2, which are the terminal effectors of the pathway. Once phosphorylated, ERKs translocate from the cytoplasm into the nucleus, where they phosphorylate a wide array of nuclear substrates, including key transcription factors such as c-Fos and c-Jun—components of the activator protein-1 (AP-1) complex [19,20]. AP-1 regulates gene expression by binding to specific DNA sequences within promoter regions of target genes involved in cell cycle progression, survival, angiogenesis, and epithelial-mesenchymal transition [13,14].

## 3. Accessory and Scaffold Proteins in the RAS/MAPK Signaling Pathway

**Figure 2:**
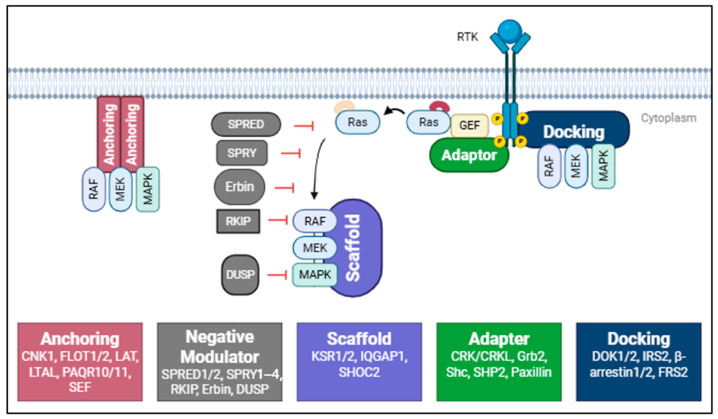
Schematic illustration of accessory and scaffold proteins regulating the RAS/MAPK signaling cascade. A variety of non-enzymatic proteins spatially and temporally organize the assembly and activity of pathway components of RAS/MAPK signaling. Anchoring, scaffold, adapter, and docking proteins coordinate the efficient activation of RAS/MAPK signaling pathway, whereas negative modulators act to attenuate the signal (see text for details).

The RAS/MAPK signaling pathway is not simply a linear cascade of kinase activation; rather, it is a highly regulated signaling network orchestrated by numerous non-enzymatic proteins that spatially and temporally coordinate signaling dynamics. Among these, accessory and scaffold proteins play indispensable roles [21,22]. Though they are not catalytic components of the pathway, they serve as molecular integrators that assemble multi-protein complexes, regulate subcellular localization, and fine-tune the magnitude, duration, and specificity of signal propagation. These proteins ensure that signaling responses are precisely modulated in accordance with extracellular stimuli and the physiological context. Accessory proteins in the RAS/MAPK pathway can be broadly categorized into four functional groups based on their structural characteristics and regulatory mechanisms: anchoring proteins, docking proteins, adaptor proteins, and scaffold proteins [4,21]. Each class contributes to organizing and orchestrating signal flow at distinct cellular compartments and stages of the cascade (Figure 2).

### 3.1. Anchoring Proteins: Spatial Regulation of Kinase Activity

Anchoring proteins primarily regulate the spatial distribution of signaling complexes by tethering pathway components to specific membrane domains or organelles. These proteins enable the spatial compartmentalization of signaling, which is increasingly recognized as a critical determinant of signaling specificity. CNK1 (Connector Enhancer of KSR1) is a well-characterized anchoring protein that supports RAF membrane recruitment and dimerization upon RAS activation. CNK1 interacts with RAF isoforms and is required for efficient RAF activation. Notably, CNK1 contains a pleckstrin homology (PH) domain that facilitates membrane association. Pharmacological inhibition of CNK1 using small molecules such as PHT-7.3 impairs KRAS4B-driven signaling by disrupting CNK1-RAS colocalization at the plasma membrane, thereby reducing downstream RAF/MEK/ERK activity [4,21,23]. Flotillins (FLOT1 and FLOT2) are lipid raft-associated proteins that localize to membrane microdomains via their prohibitin homology (PHB) domains. Flotillins mediate clustering of receptors such as EGFR and are directly implicated in enhancing MAPK pathway signaling by interacting with CRAF, MEK, and ERK following stimulation. Their function underscores the importance of membrane microdomain composition in modulating signaling efficiency [21,24,25]. LAT (Linker for Activation of T cells) and NTAL (Non–T cell Activation Linker) are transmembrane scaffold proteins involved in immune cell signaling but also participate in MAPK regulation by anchoring receptor complexes to the membrane and facilitating the recruitment of cytoplasmic signaling proteins [4,21,26].

Spatial regulation also occurs at intracellular sites. For example, PAQR10 and PAQR11, members of the progestin and adipoQ receptor family, localize RAS to the Golgi apparatus via their N-terminal cytoplasmic tails. This non-canonical localization of RAS is functionally significant, as Golgi-localized RAS can engage distinct downstream effectors compared to membrane-localized RAS. Likewise, SEF interacts with phosphorylated MEK on the Golgi and modulates MAPK activity, potentially shaping sustained ERK signaling that is critical for certain cellular outcomes [27,28,29].

### 3.2. Docking Proteins: Bridging Receptors and Signaling Modules

Docking proteins act as platforms that physically link activated receptors to intracellular signaling components. They typically interact with both membrane-localized receptors and cytoplasmic effectors, allowing rapid assembly of signaling complexes. DOK1 and DOK2 (Downstream of Kinase proteins) function in RTK signaling by recruiting RAS effectors and modulating RAS activation [30]. They contain PH and PTB (phosphotyrosine-binding) domains that confer membrane localization and enable specific interactions with phosphotyrosine residues on activated receptors. IRS2 (Insulin receptor substrate 2), a classical docking protein in insulin/IGF signaling, also plays roles in RAS/MAPK activation by facilitating the recruitment of Grb2. Its dual-domain architecture supports both membrane localization and interaction with activated RTKs [21,31].

β-arrestins 1 and 2, traditionally known for their roles in GPCR desensitization and internalization, also serve as docking proteins for MAPK signaling. Upon GPCR activation, β-arrestins can recruit and stabilize RAF-MEK-ERK complexes, allowing signal propagation not only at the plasma membrane but also from endosomal compartments. This endosomal signaling allows for sustained ERK activation and can influence specific transcriptional outcomes [21,32,33]. FGF Receptor Substrate 2 (FRS2) is a docking protein that binds activated FGFRs via its PTB domain. FRS2 becomes tyrosine-phosphorylated upon receptor stimulation and subsequently recruits adapter proteins GRB2 and SHP2 (Src homology region 2 domain-containing phosphatase-2), forming a critical link between activated RTKs and the RAS/MAPK signaling axis [4,34].

### 3.3. Adaptor Proteins: Signal Integration and Branching

Adaptor proteins function as molecular bridges, bringing together upstream activators and downstream effectors. Their modular domains allow combinatorial interactions, enabling signal branching and integration [35]. CRK and CRK-like protein (CRKL) contain an N-terminal SH2 domain followed by two SH3 domains (SH3n and SH3c). These proteins link phosphotyrosine motifs on activated receptors to various proline-rich effectors. Interestingly, the SH2 domain of CRK promotes oncogenic transformation, whereas its SH3 domains can exert context-dependent negative regulatory effects [36]. GRB2 is one of the most critical adaptors in RAS signaling. Its SH2 domain binds to phosphotyrosine residues on RTKs or docking proteins such as FRS2 and SHC (Src Homology and Collagen), while its two SH3 domains interact with proline-rich regions of SOS1 (a guanine nucleotide exchange factor), enabling the activation of RAS. GRB2 is structurally organized with two α-helices flanking a central β-sheet in the SH2 domain, providing specificity for phosphorylated motifs [37,38,39].

SHC acts upstream of GRB2 and facilitates its recruitment to receptor complexes. SHC possesses a CH2-PTB-CH1-SH2 topology and undergoes phosphorylation at multiple sites following receptor activation, acting as a versatile hub that bridges diverse upstream signals to the RAS pathway [40]. SHP2 is a protein tyrosine phosphatase with two SH2 domains and a catalytic domain that is autoinhibited via intramolecular interactions. Upon phosphorylation at Y542 by RTKs, this inhibition is relieved, and SHP2 becomes active. SHP2 plays a key positive role in RAS activation by promoting GRB2-SOS complex assembly and counteracting inhibitory phosphatases [41]. Paxillin is a focal adhesion adapter containing binding sites for SH2 and SH3 domain-containing proteins. It forms complexes with kinases and scaffold proteins involved in RAS/MAPK signaling, contributing to cytoskeletal dynamics and cell migration in a signaling-dependent manner [42].

### 3.4. Scaffold Proteins: Assembling and Modulating the MAPK Module

Scaffold proteins coordinate signaling by physically assembling multiple kinases into pre-formed complexes. This organization enhances the efficiency and specificity of signal propagation while also preventing inappropriate cross-talk. KSR1 and KSR2 are prototypical RAS/MAPK scaffolds that organize RAF, MEK, and ERK into signaling-competent complexes [43,44]. KSR1 facilitates MEK phosphorylation by RAF and promotes ERK activation, thereby acting as a positive regulator of MAPK output. Its functional homolog KSR2 performs similar roles, though it is more restricted in tissue distribution. Importantly, both KSR1 and KSR2 interact with 14-3-3 proteins, which modulate their subcellular localization and scaffold activity, thereby fine-tuning pathway dynamics [45].

IQGAP1, a multifunctional scaffold, interacts with RTKs such as EGFR and HER2 (Human Epidermal growth factor Receptor 2). It links receptor activation to the MAPK cascade either through direct interaction or via intermediate adaptors like ShcA. IQGAP1 binds both inactive and activated receptors and contributes to receptor trafficking, signal duration, and ERK nuclear translocation [46]. SHOC2 represents another important scaffold. It forms a complex with the catalytic subunit of protein phosphatase 1 (PP1) to mediate RAF dephosphorylation at Ser259, an essential step for RAF dimerization and full activation [47]. Through this function, SHOC2 acts as a gatekeeper of RAS/MAPK pathway activation and has been implicated in both developmental disorders (Noonan-like syndromes) and cancer [48].

### 3.5. Negative Modulators of RAS/MAPK Signaling

SPRED (Sprouty-related EVH1 domain-containing protein) promotes the function of RasGAPs (Ras GTPase activating proteins), thereby accelerating RAS inactivation and suppressing RAS/MAPK signaling [49]. Expression of SPRED proteins, SPRED1 in particular, is frequently reduced in various human cancers, compared with adjacent non-tumorous tissues [49,50]. Sprouty family (SPRY1–4) is a feedback inhibitor of receptor tyrosine kinase (RTK) signaling. The precise molecular mechanism by which Spry proteins restrain RTK signaling remains unclear; however, it has been proposed that they sequester GRB2 from SOS by binding to its SH2 domain, thereby impairing RAS activation [51]. RKIP (Raf Kinase Inhibitory Protein) is a prototypical negative regulator of RAS/MAPK signaling. RKIP binds to the N-terminal region of RAF, competitively blocking the RAF–MEK interaction, thereby preventing MEK phosphorylation and downstream ERK activation [52]. Beyond this direct inhibitory role, RKIP functions as a signaling switch: under certain stimuli, protein kinase C (PKC)–mediated phosphorylation of RKIP induces its dissociation from RAF, relieving inhibition and allowing MAPK signaling to proceed [53]. Loss or downregulation of RKIP has been observed in various cancers, including prostate, breast, and hepatocellular carcinoma, where it correlates with enhanced metastatic potential and poor prognosis, underscoring its role as a metastasis suppressor [54,55,56,57].

Erbin, a LAP (leucine-rich repeat and PDZ domain) family protein, also participates in fine-tuning RAS/MAPK signaling activity (Table 1). Erbin directly binds the C-terminal tail of HER2 and, through its interaction with SHOC2, regulates the activation status of RAF. As well, by disrupting the interaction between RAS and RAF, Erbin can dampen RAS/MAPK pathway activation [58]. Dual-Specificity Phosphatases (DUSPs) not only directly dephosphorylate ERK, but they also physically interact with RAS/MAPK components to tether them in inactive complexes [59,60]. DUSP6 (MKP-3), for instance, preferentially targets ERK1/2 and has been reported to restrain MAPK-driven proliferation in multiple tissues [61]. Reduced expression of DUSP7 has been observed in cervical cancer, permitting sustained MAPK activation [62].

## 4. KSR: A Central Scaffold in RAS/MAPK Signaling

**Figure 3:**
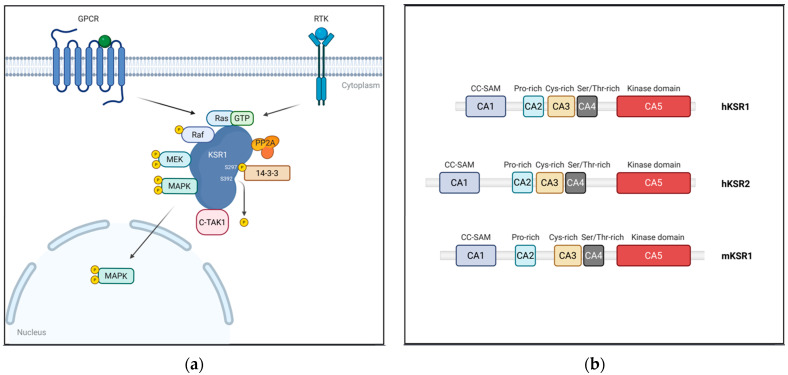
Involvement of kinase suppressor of Ras 1 (KSR1) in RAS/MAPK pathway activation and structural organization of KSR proteins. (**a**) KSR1 functions as a scaffold protein that facilitates the assembly of RAF, MEK, and ERK, thereby promoting activation of the RAS/MAPK signaling cascade and driving oncogenic signaling. (**b**) Schematic representation of the conserved domain architecture of KSR proteins. Both human (hKSR1, hKSR2) and murine (mKSR1) KSR share five conserved domains (CA1–CA5), which mediate interactions with signaling partners and regulate pathway activity.

The Kinase Suppressor of Ras (KSR) represents a unique class of regulatory proteins that function predominantly as molecular scaffolds within the RAS/MAPK signaling pathway. Initially identified through classical genetic screens in Drosophila melanogaster and Caenorhabditis elegans, KSR was discovered not as a conventional kinase but rather as a modulator essential for Ras-driven phenotypes [64,65]. This finding shifted the paradigm of signal transduction from a sole emphasis on enzymatic phosphorylation events to a broader understanding of the critical role played by scaffolding proteins. Unlike traditional kinases that catalyze phosphorylation, KSR proteins exhibit limited intrinsic kinase activity. Instead, KSR exerts its regulatory function by physically assembling the core components of the RAS/MAPK cascade—RAF, MEK, and ERK—into macromolecular complexes (Figure 3a). This assembly not only increases the efficiency of signal transmission but also ensures signaling specificity by restricting cross-talk and inappropriate pathway activation. Through its modular domain structure and tightly regulated subcellular localization, KSR acts as a precision controller that modulates the amplitude, duration, and localization of MAPK signaling outputs, thereby influencing diverse cellular outcomes such as proliferation, differentiation, and survival.

### 4.1. Structure of KSR Proteins

In mammals, two paralogs of the Kinase Suppressor of Ras (KSR) have been identified, namely KSR1 and KSR2, which share significant structural similarity. Both KSR1 and 2 proteins exhibit a domain organization reminiscent of RAF kinases. This shared architecture consists of five conserved regions, termed CA1 through CA5, each contributing distinct functional roles critical for KSR’s scaffolding activity and regulatory control within the MAPK pathway (Figure 3b) [44,69].

#### 4.1.1. CA1 (Coiled-Coil/SAM Domain)

Located at the N-terminus, the CA1 domain encompasses approximately 40 amino acids, forming a sterile alpha motif (SAM). This domain is essential for membrane association and heterodimerization with RAF kinases, with a particular affinity for B-RAF. The SAM domain facilitates the spatial organization of signaling complexes at the plasma membrane, an essential step during pathway activation, thereby enabling effective signal transmission [70,71].

#### 4.1.2. CA2 (Proline-Rich Domain)

Although less characterized, the proline-rich CA2 domain likely mediates interactions with SH3 domain-containing adaptor or signaling proteins. The region adjacent to CA2 has been shown to interact with AMP-activated protein kinase (AMPK), suggesting a novel role for KSR in integrating cellular energy status with MAPK signaling output, potentially linking metabolic cues to proliferative signaling [72].

#### 4.1.3. CA3 (Atypical C1 Domain)

Homologous to the CR1 domain of RAF kinases, the domain diverges functionally by lacking robust diacylglycerol (DAG) binding. Instead, it retains the capacity to bind negatively charged phospholipids, anchoring KSR to anionic lipid-enriched microdomains at the plasma membrane. This membrane tethering is critical for colocalizing MEK and RAF kinases within a confined membrane environment, facilitating efficient phosphorylation events [73].

#### 4.1.4. CA4 (Serine/Threonine-Rich Domain)

This region contains an FXFP motif, a canonical docking site recognized by activated ERK. By retaining ERK within the scaffolded complex, KSR regulates the kinetics and localization of ERK activation. Moreover, this facilitates feedback phosphorylation of KSR itself or other scaffold components by ERK, thus modulating ERK substrate specificity and fine-tuning downstream signaling responses [44].

#### 4.1.5. CA5 (Kinase/Pseudokinase Domain)

Structurally analogous to the CR3 kinase domain of RAF, CA5 is notable for its ability to bind MEK despite lacking robust kinase activity. Although KSR has been known as a pseudokinase, emerging evidence reveals that KSR can exhibit context-dependent catalytic function. Mutations or substitutions within CA5 have been shown to severely impair RAS/MAPK pathway activation, underscoring the critical scaffold-kinase interface [44,74].

### 4.2. Dynamic Regulation of KSR1 Localization

KSR1 is regulated by a multilayered mechanism involving phosphorylation events, interactions with regulatory proteins, and controlled intracellular trafficking, collectively ensuring signaling precision. Under resting conditions, C-TAK1 kinase phosphorylates KSR1 at serine residues Ser297 and Ser392. These phosphorylations promote high-affinity binding of 14-3-3 proteins, resulting in the cytosolic sequestration of KSR1 in an inactive conformation, thus preventing unwarranted MAPK activation [45]. Upon extracellular stimuli such as growth factor binding, activation of upstream RAS triggers protein phosphatase 2A (PP2A) to dephosphorylate Ser392. This dephosphorylation disrupts the 14-3-3 interaction, enabling KSR1 to translocate to the plasma membrane. At the membrane, KSR1 assembles the RAF–MEK–ERK signaling module into an active complex, thereby potentiating signal transduction [45].

The regulation of KSR1 localization restricts KSR1′s scaffolding function to appropriate signaling contexts, preventing aberrant RAS/MAPK pathway activation. Importantly, the subcellular localization of KSR1 is a critical determinant of RAS/MAPK pathway dynamics: membrane-associated KSR1 facilitates rapid and robust ERK activation, whereas cytoplasmic retention dampens signaling intensity and duration.

### 4.3. Beyond Scaffolding: Alternative Functions of KSR1 in RAS/MAPK Signaling

Traditionally, KSR1 is a major scaffold protein of the RAS/MAPK signaling pathway, forming a complex with the major components and facilitating phosphorylation events among them. For example, by simultaneously binding RAF and MEK, KSR1 spatially aligns these kinases and enhances MEK phosphorylation by RAF. In addition to the scaffolding role, several alternative mechanisms have been proposed on how KSR1 amplifies and modulates RAS/MAPK pathway signaling.

Research from Therrien and colleagues demonstrated that MEK binding to the kinase domain of KSR1 promotes asymmetric heterodimerization with BRAF, which allosterically enhances BRAF catalytic activity toward unbound MEK substrates [75]. These findings indicate that KSR–MEK complexes can allosterically activate BRAF via interactions between KSR’s N-terminal regulatory region and kinase domain, challenging the conventional view of KSR solely as a scaffold for recruiting MEK to RAF. As well, KSR1 likely protects MEK and ERK from dephosphorylation, extending active signaling—consistent with scaffold-mediated insulation of kinases and the prolonged ERK activation [76].

## 5. Roles of KSR1 in Cancer

**Figure 4:**
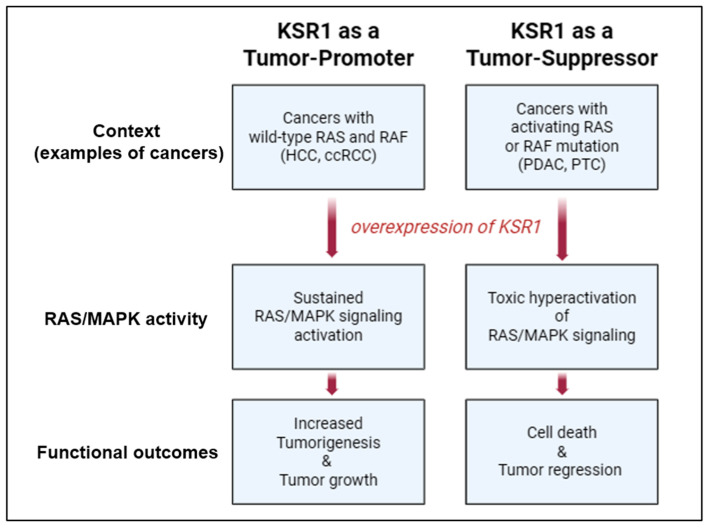
Dual roles of KSR1 in cancer. KSR1 can exert either tumor-promoting or tumor-suppressive effects depending on the cellular and genetic context. KSR1 overexpression facilitates RAS/MAPK pathway activation that supports proliferation in many cancers; however, KSR1 overexpression may lead to hyperactivation of RAS/MAPK signaling in cancers harboring active *RAS* or *RAF* mutations, resulting in growth arrest or apoptosis.

Kinase suppressor of Ras 1 (KSR1) occupies a unique position within the RAS/MAPK signaling network, serving as both a facilitator and, under certain circumstances, a restrainer of pathway output. This functional duality—tumor-promoting in some contexts, tumor-suppressive in others—presents both a conceptual challenge and an opportunity for therapeutic innovation. While most experimental evidence underscores KSR1′s role in scaffolding RAF, MEK, and ERK to enhance oncogenic signaling, a subset of studies demonstrates that excessive KSR1 expression can paradoxically attenuate RAS/MAPK activity [77]. This duality in KSR1 function highlights its complexity as a signaling regulator. The same molecular scaffold can either enhance or restrain oncogenic signaling, depending on dosage, post-translational modifications, interaction partners, and cellular context. For therapeutic development, such biphasic behavior underscores the importance of precisely modulating KSR1 activity (Figure 4).

### 5.1. KSR1 as a Tumor-Promoter

Accumulating evidence from diverse experimental models indicates that KSR1 functions as a tumor-promoter, largely through its role in facilitating persistent activation of the RAS/MAPK signaling cascade. Functional analyses in mouse embryonic fibroblasts (MEFs) have provided direct evidence for KSR1′s oncogenic potential. Ectopic expression of KSR1 in MEFs induced sustained ERK phosphorylation, accelerated S-phase entry, and enhanced cell proliferation [78]. Conversely, KSR1-deficient MEFs displayed attenuated ERK activation and impaired transformation, phenotypes that were restored in a dose-dependent manner upon KSR1 re-expression, with ERK activation and transformation increasing up to 14-fold [76]. Notably, in Ras-deficient MEFs, ectopic KSR1 expression restored colony-forming ability, underscoring KSR1′s capacity to drive RAS/MAPK pathway activation independently of upstream RAS activity [79].

Loss-of-function studies in various cancer contexts further support KSR1′s tumor-promoting role. Targeted knockdown of KSR1 using shRNA markedly impairs anchorage-independent growth in soft agar assays and reduces tumor formation in xenograft mouse models, confirming its role in promoting tumorigenesis [69,80]. In pancreatic cancer, KSR1 deletion decreased RAS/MAPK signaling, suppressed cellular transformation, and modestly prolonged survival in mouse models [81]. In hematologic malignancies, KSR1 deficiency attenuated Myc activation via the RAS/MAPK pathway, reduced B cell proliferation, increased apoptosis, and delayed lymphoma onset [82]. In melanoma, KSR1 deletion impaired ERK phosphorylation, leading to reduced proliferation, invasion, and metastasis, along with increased senescence and apoptosis [83]. In skin tumor models, KSR1 deletion alone did not abolish papilloma formation, but significantly reduced tumorigenesis in the presence of oncogenic Ras mutations [84]. Similarly, KSR1 knockout mice displayed a significant reduction in mammary tumor burden in models expressing the polyomavirus middle T antigen, indicating the in vivo tumor-promoting function of KSR1 [85]. Taken together, these findings firmly establish KSR1 as a pivotal amplifier of oncogenic RAS/MAPK signaling. By modulating the intensity and persistence of ERK activation, KSR1 influences the threshold for tumor initiation and progression

### 5.2. KSR as a Tumor-Suppressor

Although the name Kinase Suppressor of Ras (KSR1) historically suggested an inhibitory role in RAS-mediated oncogenesis, evidence for its tumor-suppressive function is comparatively limited and context-dependent. Early studies reported that murine KSR1 could antagonize oncogenic Ras activity. In NIH3T3 cells, KSR1 expression suppressed Ras-induced transformation, while in embryonic neuroretina cells, it inhibited proliferation [86]. These findings provided the first experimental support for a potential negative regulatory function. In human embryonic kidney cells stimulated with insulin or phorbol ester—both potent activators of the RAS/MAPK pathway through protein kinase C—ectopic expression of KSR1 attenuated pathway activation [87,88].

Clinical evidence also points to a potential tumor-suppressive role for KSR1 in certain contexts. In breast cancer, higher KSR1 expression correlated with improved patient survival [89], suggesting that in specific tumor settings, KSR1 may attenuate rather than enhance oncogenic RAS/MAPK signaling. Supporting this notion, KSR1 expression has been reported to be significantly reduced in pancreatic ductal adenocarcinoma (PDAC) and papillary thyroid carcinoma (PTC) compared with adjacent non-tumorous tissue [90]. In contrast, hepatocellular carcinoma (HCC) and clear cell renal cell carcinoma (ccRCC) display marked upregulation of KSR1. It is noteworthy that activating mutations in the RAS and RAF genes are found in more than 90% of PDAC [91,92,93] and 70% of PTC cases [94], respectively, whereas such mutations are rarely detected in HCC and ccRCC.

The mechanisms underlying KSR1 downregulation in PDAC and PTC remain unclear, but one possibility is that reduced KSR1 expression serves to limit excessive RAS/MAPK pathway activation in tumors already driven by RAS or RAF mutations. Recent studies have demonstrated that hyperactivation of the RAS/MAPK cascade can be detrimental to tumor cell survival [95,96]. For example, enforced ERK expression in melanoma cells carrying an activating mutation in BRAF (BRAF^V600E^) triggered cell death in vitro and tumor regression in xenograft models [95]. Similarly, colorectal cancer cell lines harboring KRAS or BRAF mutations exhibited marked sensitivity to PP2A inhibition, which caused further amplification of MAPK signaling and led to cell death [96]. Together, these findings raise the intriguing possibility that elevated KSR1 expression may function as a tumor suppressive mechanism in cancers harboring constitutively active RAS or RAF mutations by driving RAS/MAPK signaling into a state of toxic hyperactivation, whereas in tumors lacking such mutations (e.g., HCC, ccRCC), increased KSR1 levels instead enhance oncogenic signaling and promote tumorigenesis.

## 6. Targeting KSR1

Although KSR1 has been well-characterized as a scaffold protein that coordinates RAS/MAPK cascade activation, its potential as a therapeutic target in oncology remains underexplored.

### 6.1. Preclinical Studies Targeting KSR1

Preclinical studies directly investigating KSR1 as a therapeutic target remain limited, largely due to the lack of potent and selective KSR1 inhibitors. Nevertheless, several lines of evidence suggest that KSR1 plays a critical role in tumor progression and may represent a tractable vulnerability. In endometrial cancer cell lines, KSR1 knockdown reduced ERK phosphorylation and suppressed cell proliferation [97]. Similarly, siRNA-mediated silencing of KSR1 in colorectal cancer cells triggered apoptosis and inhibited tumor growth in xenograft models [80]. In melanoma harboring the oncogenic BRAF^V600E^ mutation, KSR1 knockout impaired cell proliferation, induced cell cycle arrest, and promoted apoptosis, highlighting a context-dependent synthetic vulnerability in BRAF-driven tumors [83]. Collectively, these findings point to KSR1 as a potentially valuable therapeutic target across multiple tumor types. However, the current evidence base is limited, emphasizing the urgent need for the development of effective KSR1-directed inhibitors, as well as optimized delivery strategies to achieve therapeutic efficacy in vivo.

While most current efforts focus on KSR1 inhibition, it is also conceivable that enhancing KSR1 activity could yield therapeutic benefits in specific tumor contexts where KSR exerts tumor-suppressive effects. Although it is theoretically possible to boost KSR’s activity in cancer via, for example, delivering a viral vector expressing KSR1, targeted activation of KSR1 remains technically challenging and untested in cancer. Moreover, given the context-dependent and potentially oncogenic consequences of KSR1 activation, current translational strategies are more feasibly directed toward KSR1 inhibition.

### 6.2. Potential Strategies for KSR1 Targeting

**Figure 5:**
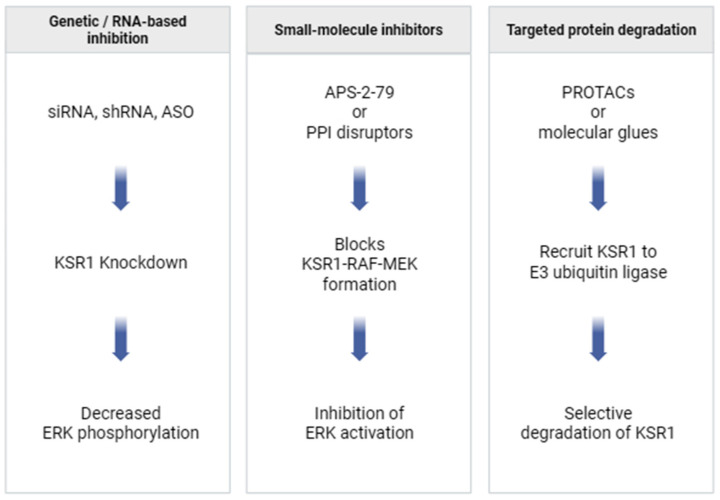
Potential strategies for KSR1 targeting. KSR1 activity in cancer can be therapeutically suppressed through multiple approaches, including RNA interference or antisense oligonucleotides, small-molecule inhibitors that disrupt KSR1–RAF/MEK interactions, and targeted protein degradation strategies such as PROTACs.

Given its central role as a scaffold in the RAS/MAPK cascade, KSR1 presents a unique set of opportunities and challenges for therapeutic targeting. Unlike kinases with well-defined catalytic pockets, KSR1 primarily functions through protein–protein interactions (PPIs) with upstream and downstream effectors, including RAF, MEK, and ERK. This structural role necessitates innovative strategies that disrupt its scaffolding function without broadly impairing MAPK signaling required for normal physiology (Figure 5).

#### 6.2.1. Small-Molecule Disruptors of KSR1–RAF/MEK Interactions

Structural studies have identified multiple domains within KSR1 that mediate its binding to RAF kinases and MEK. Small molecules or peptidomimetics that selectively block these interfaces could effectively uncouple KSR1 from the MAPK module, reducing signal amplification in oncogenic contexts. Although no clinically approved compounds exist, high-throughput screening for PPI inhibitors has yielded candidate molecules capable of displacing KSR1 from RAF in vitro, leading to attenuated ERK phosphorylation. One of the most significant advances in the pharmacological targeting of KSR has been the discovery of APS-2-79, a small-molecule allosteric inhibitor that interferes with KSR and RAF heterodimerization, and inhibits oncogenic RAS/MAPK signaling [98]. By targeting a specific pocket within the pseudokinase domain, APS-2-79 stabilizes KSR in an inactive conformation, thereby preventing the formation of a functional RAF–KSR–MEK complex [69].

APS-2-79 demonstrated only modest activity in reducing cell viability in RAS-mutant cancer cell lines and showed little to no effect in RAF-mutant cancer cells [98]. This limited efficacy contrasts with the more pronounced tumor-suppressive effects observed following genetic depletion of KSR1, where siRNA- or knockout-mediated suppression of KSR1 markedly impaired cell proliferation, induced apoptosis, and reduced tumor growth in both in vitro and in vivo models [80,83,97]. These findings highlight an important gap between genetic validation of KSR1 as a therapeutic target and the current pharmacological tools available to inhibit its function. Consequently, there is a pressing need for the development of next-generation KSR1 inhibitors with improved potency and pharmacokinetic properties.

#### 6.2.2. Targeted Protein Degradation Approaches

Targeted protein degradation strategies such as PROTACs (proteolysis-targeting chimeras) or molecular glues may be effective [99,100,101]. By recruiting KSR1 to E3 ubiquitin ligases, these agents could selectively deplete the protein in tumor cells while sparing non-essential paralogs (e.g., KSR2) or unrelated RAS/MAPK components.

#### 6.2.3. RNA-Based Silencing Methods

RNA interference (RNAi) or antisense oligonucleotide (ASO) strategies have already demonstrated preclinical efficacy in suppressing KSR1 expression. siRNA-mediated knockdown has been shown to reduce proliferation, impair ERK signaling, and induce apoptosis in multiple tumor cell types, as noted above. Such approaches may be especially suitable for localized delivery to tumors, where nanoparticle-based carriers could be employed for targeted tissue uptake [102,103].

## 7. Targeting KSR1 in Hepatocellular Carcinoma

Hepatocellular carcinoma (HCC) accounts for more than 80% of primary liver cancers and ranks as the third leading cause of cancer-related death worldwide [104,105]. Global incidence is projected to rise in the coming decade, driven by persistent risk factors such as chronic hepatitis B and C infection, alcohol-related liver disease, and the growing prevalence of metabolic-associated steatohepatitis [106,107,108].

One particularly intriguing feature of hepatocellular carcinoma (HCC) is that activating mutations in *RAS* and *RAF* genes—common oncogenic drivers in many other solid tumors—are rare. Despite this low mutational frequency, the RAS/MAPK signaling pathway is frequently hyperactivated in HCC, with more than half of cases exhibiting elevated ERK activity [109,110]. This paradox suggests that pathway activation in HCC is largely driven by alternative mechanisms, including overexpression or constitutive activation of upstream receptor tyrosine kinases (such as EGFR, FGFR, and c-Met), amplification of autocrine or paracrine growth factor signaling, and suppression of negative regulators like Sprouty and dual-specificity phosphatases (DUSPs). These non-mutational mechanisms converge to sustain RAS/MAPK pathway signaling, promoting tumor cell proliferation, survival, invasion, and therapeutic resistance, and they underscore the importance of targeting pathway modulators beyond the canonical mutational hotspots.

### 7.1. Preclinical Studies Targeting RAS/MAPK Signaling in HCC

RAS/MAPK signaling is frequently upregulated in hepatocellular carcinoma (HCC). Comparative analyses have shown that in more than 50% of HCC tissues, RAS/MAPK activity is elevated relative to adjacent non-malignant liver tissues [13]. Pharmacologic inhibition of RAS/MAPK signaling with rigosertib suppresses HCC cell proliferation and induces apoptosis, accompanied by marked decreases in ERK phosphorylation [111,112]. However, the precise downstream mechanisms of rigosertib-mediated RAS inhibition remain incompletely understood. At the RAF node, RAF1 is directly targeted by miR-4510, leading to ERK inactivation and downregulation of ERK-dependent genes. Silencing RAF1 with siRNA similarly reduces ERK activity and proliferation in HCC cells [113].

MEK inhibition has also demonstrated antitumor efficacy in preclinical HCC models. The MEK inhibitor cobimetinib significantly reduced proliferation and induced apoptosis in sorafenib-resistant Hep3B cells. In xenograft mice bearing sorafenib-resistant HCC, cobimetinib treatment suppressed ERK phosphorylation and slowed tumor progression [114]. Likewise, siRNA-mediated knockdown of ERK2 in rat HCC cells reduced DNA synthesis, and pharmacologic inhibition with the MEK inhibitor U0126 produced comparable reductions in proliferative cell populations [115].

In addition to the core signaling kinases, several accessory proteins modulate RAS/MAPK signaling in HCC. The adaptor protein GRB2 links receptor tyrosine kinases to RAS activation via SOS [116]. Overexpression of RNF173 promotes GRB2 degradation, thereby suppressing RAF/MEK/ERK signaling and reducing HCC malignancy [117]. Another adaptor, SHP2, facilitates RAS/MAPK activation [118,119]. SHP2 knockdown in HCC cells markedly reduces tumorigenic potential, whereas reintroduction of wild-type SHP2 restores proliferation and tumor growth. In xenograft models, SHP2 silencing significantly suppressed tumor formation, while SHP2 overexpression accelerated tumor progression [120].

Negative regulators of RAS/MAPK signaling also play critical roles in HCC. SPRED1 overexpression suppresses ERK activation and proliferation, whereas dominant-negative SPRED1 mutants or SPRED1-targeting miRNAs enhance ERK phosphorylation [121,122]. In xenograft models, SPRED1 expression attenuated ERK activity, particularly when combined with sorafenib treatment. Similarly, SPRED2 overexpression inhibited proliferation and induced apoptosis, while SPRED2 knockdown increased ERK phosphorylation and accelerated tumor growth in vivo [123,124]. Another negative regulator, RKIP, binds RAF to block RAS/MAPK signaling [125]. In HCC cells, RKIP expression inversely correlates with MEK/ERK phosphorylation, and RKIP overexpression reduces HCC cell proliferation and migration by suppressing IGF-I–mediated MAPK activation [126].

Collectively, these findings underscore the central role of RAS/MAPK signaling in HCC pathogenesis and highlight multiple nodes—including RAF, MEK, ERK, adaptor proteins, and regulators—that represent viable targets for therapeutic intervention (Table 2).

### 7.2. Resistance to Drugs Targeting the RAS/MAPK Signaling Pathway in HCC

Although considerable progress has been made in developing targeted therapies for hepatocellular carcinoma (HCC), their long-term clinical efficacy remains limited due to the emergence of drug resistance [127,128,129,130]. Such resistance can be intrinsic—present before treatment initiation—or acquired during therapy as tumor cells adapt to sustained drug pressure [131]. Consequently, most patients with advanced HCC fail to achieve durable responses to systemic therapies, highlighting the urgent need to better understand and overcome resistance mechanisms.

Multiple mechanisms have been identified by which HCC cells evade the tumoricidal effects of molecularly targeted therapies. For example, inhibition of fibroblast growth factor receptor (FGFR) by the treatment with lenvatinib can trigger activation of the EGFR–PAK2–ERK5 signaling axis [128,132,133]. Accordingly, combined treatment with the EGFR inhibitor gefitinib and lenvatinib exerts strong anti-proliferative effects in EGFR-expressing liver cancer cell lines in vitro, as well as in xenografted cell lines, immunocompetent mouse models, and patient-derived HCC xenografts in vivo [134]. Using a CRISPR-based library screen, Lu et al. identified NF1 and DUSP9 as key drivers of lenvatinib resistance in HCC and further demonstrated that NF1 loss reactivates both the PI3K/AKT and RAS/MAPK pathways, whereas DUSP9 loss selectively activates the RAS/MAPK pathway, collectively promoting resistance [135].

Several mechanisms have been implicated in sorafenib resistance in HCC. Overexpression of fibroblast growth factors (FGFs) can promote resistance, thereby sustaining cancer cell survival [136,137,138]. Similarly, activation of the hepatocyte growth factor (HGF)/c-Met signaling axis has been shown to facilitate resistance [139]. Aberrant activation of EGFR and HER3 receptors, together with overexpression of multiple EGFR ligands, has also been observed in sorafenib-resistant HCC, providing an additional mechanism for drug evasion [140]. Intriguingly, enrichment of cancer stem cells (CSCs) represents another route of resistance, as CSCs possess intrinsic survival advantages and reduced drug uptake, enabling them to withstand sorafenib-induced tumor suppression [141,142]. Additionally, compensatory activation of the PI3K/AKT pathway provides an alternative survival route, enabling HCC cells to bypass sorafenib-induced apoptosis [143]. Consistent with this, combined treatment with sorafenib and MK-2206, an Akt inhibitor, has been shown to overcome the emergence of sorafenib resistance [144].

Regorafenib has been established as a second-line treatment option for advanced HCC following failure of first-line sorafenib or lenvatinib therapy [145,146,147,148,149]. However, in vitro studies have shown that long-term exposure to regorafenib can lead to the emergence of regorafenib-resistant HCC cells characterized by enhanced TGF-β signaling activity [150]. Inhibition of the TGF-β receptor in combination with regorafenib significantly increased cancer cell death and senescence. In another study, regorafenib-resistant HCC cells exhibited high expression of SphK2. Ectopic overexpression of SphK2 reduced sensitivity to regorafenib via concomitant activation of nuclear factor κB (NF-κB) and signal transducer and activator of transcription 3 (STAT3) signaling pathways [151]. Collectively, these findings highlight the multifaceted mechanisms by which HCC cells develop resistance to current RTK- and RAS/MAPK-targeted therapies and emphasize the need for novel strategies to overcome resistance within this signaling network (Table 3).

### 7.3. KSR as a Therapeutic Target in HCC

The Kinase Suppressor of Ras (KSR) plays a pivotal role in regulating the RAS/MAPK signaling cascade [69,79]. Overexpression of KSR has been shown to directly activate MAPK signaling [79] and to contribute to MAPK reactivation and ERK phosphorylation following pharmacologic inhibition of BRAF or MEK [152]. Furthermore, elevated KSR1 expression has been linked to reduced sensitivity to KRAS inhibitors [79]. In several malignancies—including pancreatic ductal adenocarcinoma, melanoma, and skin carcinoma—KSR1 has been identified as an oncogenic facilitator of RAS/MAPK signaling, and its genetic or pharmacological inhibition suppresses tumor growth [79]. In contrast, studies investigating KSR1 in hepatocellular carcinoma (HCC) have been remarkably limited until recently. In HCC models, KSR2 knockdown suppresses proliferation and migration, whereas KSR2 overexpression enhances malignant phenotypes, increases MEK and ERK phosphorylation, and attenuates the growth-inhibitory effects of sorafenib [153].

The recent work by Moon et al. represents the first comprehensive study to establish a functional link between KSR1 and HCC pathogenesis [90], offering new insights into targeting RAS/MAPK signaling. KSR1 overexpression was frequently observed in HCC patient samples and strongly correlated with RAS/MAPK pathway activity. Functionally, KSR1 overexpression selectively increased pMEK1/2 and pERK1/2 levels both in vitro and in vivo, underscoring its role as a positive regulator of RAS/MAPK signaling in HCC. Importantly, overexpression of KSR1, when cooperated with p53 inactivation or c-Myc overexpression, drives efficient hepatocarcinogenesis, achieving tumorigenic potency comparable to that induced by activating mutations in *RAS* or *RAF*.

**Figure 6:**
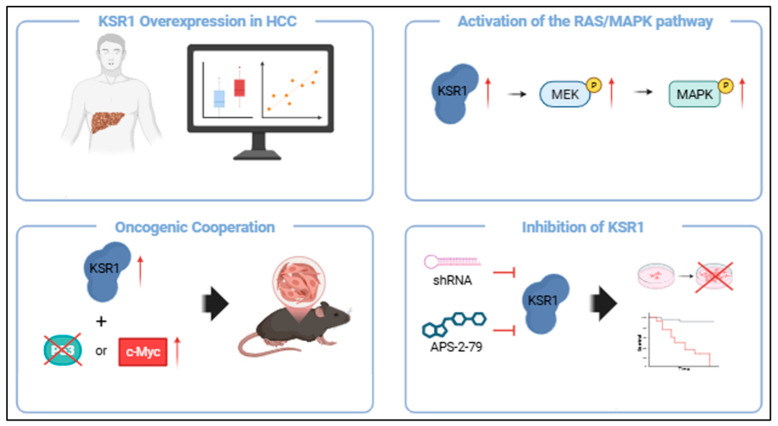
KSR1 in hepatocellular carcinoma (HCC). KSR1 is highly expressed in HCC and positively linked to RAS/MAPK activity. KSR1 overexpression leads to RAS/MAPK pathway activation via enhanced phosphorylation of MEK and ERK. In murine models, KSR1 drives hepatocarcinogenesis in cooperation with P53 inactivation or c-Myc overexpression. Conversely, inhibition of KSR1—via shRNA-mediated knockdown or pharmacological blockade using APS-2-79—attenuates RAS/MAPK pathway activation and suppresses tumor progression [90].

Intriguingly, inhibition of KSR1—either by shRNA or by APS-2-79, a pharmacological inhibitor of KSR—significantly reduced RAS/MAPK pathway activity [90]. Treatment with APS-2-79 suppressed HCC cell proliferation in vitro and prolonged survival in HCC-bearing mice. Together, these findings demonstrate that KSR1 can drive RAS/MAPK pathway activation in the liver independently of upstream *RAS* or *RAF* mutations, highlighting KSR1 as a promising therapeutic target in HCC with aberrant activation of RAS/MAPK signaling (Figure 6).

The significance of the Moon et al. study lies in its pioneering demonstration that KSR1 inhibition represents a viable therapeutic strategy for HCC, thereby broadening the spectrum of actionable nodes within the RAS/MAPK signaling network. While most prior studies of KSR1 were performed in non-hepatic cancer models, this work establishes a critical proof-of-concept in liver cancer, underscoring KSR1′s role as a potential oncogenic driver. Looking forward, this discovery is expected to stimulate further research into KSR biology in HCC, including the development of next-generation KSR1 inhibitors with improved selectivity, bioavailability, and potency. Moreover, these findings position KSR1 as an emerging molecular vulnerability in hepatocellular carcinoma, representing a novel therapeutic target.

### 7.4. Clinical Studies Targeting Other Dysregulated Elements of the RAS/MAPK Pathway

Although clinical trials targeting KSR1 have yet to be initiated, clinical studies to target the RAS/MAPK signaling cascade at multiple levels in various cancers could inform the therapeutic effect of targeting KSR1 in human cancers, including HCC. Inhibiting farnesylation—essential for RAS membrane localization and activity—initially showed promise, but farnesyltransferase inhibitors like lonafarnib yielded disappointing results in clinical trials due to compensatory prenylation by geranylgeranyl transferase I [154,155]. Consequently, downstream components of the RAS/MAPK signaling pathway have emerged as more tractable targets. Selective BRAF inhibitors, including vemurafenib and dabrafenib, have significantly improved overall and progression-free survival in patients with melanoma [156,157,158].

Notably, MEK inhibitors have shown remarkable efficacy in certain cancers. For example, selumetinib, recently approved by the FDA, demonstrated clinical benefit in relapsed low-grade serous ovarian cancer and neurofibromatosis type 1 [159,160]. As well, trametinib and cobimetinib have shown meaningful improvements in progression-free survival and overall response rates in various types of cancers [161,162].

**Figure 7:**
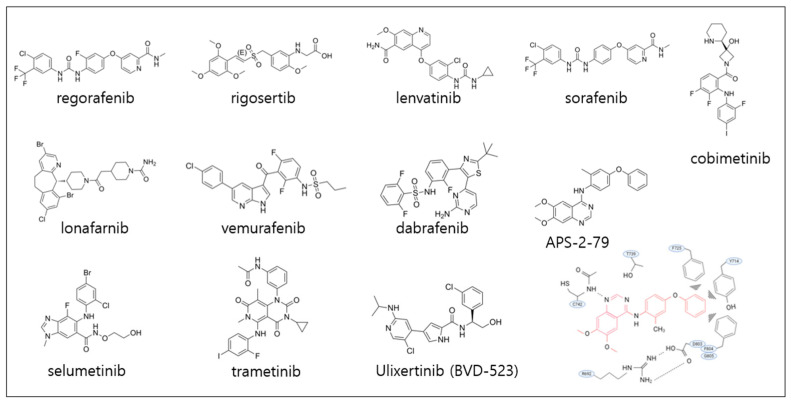
Chemical structures of small-molecule inhibitors cited in this review. The binding mode of APS-2-79 with KSR2 is also illustrated below the chemical (adapted from reference [98]).

With recognition that ERK reactivation underlies resistance to RAF or MEK inhibitors, several clinical studies have been performed by targeting ERK directly. Selective ERK1/2 inhibitors such as BVD-523 are in clinical development for RAS- or RAF-mutant tumors (Figure 7). BVD-523 has shown potent anti-proliferative activity in colorectal and pancreatic cancers harboring BRAF or KRAS mutations [9,163]. Nevertheless, adaptive resistance remains a major obstacle to durable responses with RAS/MAPK-targeted therapies. This persistent challenge underscores the need for novel therapeutic approaches that go beyond single-agent inhibition. Future strategies may include rational combination therapies integrating KSR1 inhibition with RAF, MEK, or ERK inhibitors to enhance antitumor efficacy and overcome adaptive resistance in HCC and other RAS/MAPK-driven malignancies.

## 8. Perspectives and Conclusions

KSR1 has emerged as a pivotal scaffold protein within the RAS/MAPK signaling cascade, orchestrating the spatial and temporal assembly of pathway components to ensure efficient and sustained activation of MEK and ERK [43,44]. Beyond its canonical scaffolding function, KSR1 is increasingly recognized as a dynamic regulator of signaling intensity, duration, and subcellular localization. By modulating the proximity and phosphorylation state of RAF, MEK, and ERK, KSR1 fine-tunes the signaling output of the RAS/MAPK pathway in a highly context-dependent manner [43,44]. At physiological levels, KSR1 facilitates balanced MAPK signaling that supports controlled proliferation and survival. However, its overexpression or dysregulation can amplify signaling flux, thereby promoting oncogenic transformation.

### 8.1. Targeting KSR1 in HCC

From a therapeutic standpoint, we believe that targeting KSR1 represents a conceptually distinct and underexplored strategy for modulating oncogenic RAS/MAPK signaling. By selectively destabilizing or disrupting the scaffold architecture that coordinates RAS/MAPK signaling, it may be possible to attenuate oncogenic signaling in tumor cells while preserving the physiological signaling needed in normal tissues. Thus, pharmacological manipulation of KSR1 could allow fine-tuned pathway control. In HCC, a malignancy characterized by frequent RAS/MAPK pathway activation despite the rarity of RAS or RAF mutations, KSR1 appears to serve as a non-mutational driver of oncogenic signaling. Elevated KSR1 expression in HCC correlates with enhanced MEK and ERK phosphorylation and aggressive clinical behavior, suggesting that KSR1 contributes to the constitutive activation of RAS/MAPK signaling that underpins HCC progression. Functional studies have shown that KSR1 silencing in HCC, either through RNA interference or pharmacological blockade, attenuates RAS/MAPK signaling and inhibits tumor growth both in vitro and in vivo [43,44]. Notably, KSR1-deficient mice develop normally and remain viable, implying that KSR1 may be dispensable for normal tissue homeostasis yet essential for tumor maintenance [84]. This observation strengthens the case for KSR1 as a therapeutically tractable and cancer-selective vulnerability.

The development of small molecules such as APS-2-79, which allosterically stabilize KSR1 in an inactive conformation and prevent RAF–KSR–MEK complex formation, provides proof-of-principle that KSR1 is druggable [43,44]. However, these early agents show modest potency and limited selectivity, underscoring the need for more sophisticated approaches. In our view, the next frontier for KSR1 research lies in integrating structural biology, proteomics, and chemical innovation to design compounds that more precisely modulate its scaffolding function or promote its selective degradation.

### 8.2. Future Research Directions

Several key directions warrant attention in future research. First, high-resolution structural characterization of KSR1 in its active and inactive states will be essential for rational drug design. In particular, the dynamic transitions that occur during complex formation with RAF and MEK remain poorly understood. For example, hydrogen–deuterium exchange mass spectrometry could illuminate these conformational landscapes, revealing transient pockets or surfaces amenable to allosteric inhibition [164]. Second, the development of next-generation KSR1 inhibitors should expand beyond traditional small molecules. Emerging technologies such as proteolysis-targeting chimeras (PROTACs) or molecular glues offer the potential to achieve selective and complete degradation of KSR1 in cancer cells [99,100,101]. These approaches could circumvent the limitations of occupancy-driven inhibition by eliminating the protein entirely, providing more durable pathway suppression. Parallel optimization of pharmacokinetic and delivery properties—such as liver-specific nanoparticle formulations—will be crucial for achieving effective targeting in HCC. Finally, rational combination strategies with MEK inhibitors, tyrosine kinase inhibitors, or immunotherapies may also reveal synergistic effects capable of overcoming adaptive resistance to current treatments.

### 8.3. Combination Therapies as Possible Therapeutic Strategies

Given KSR1′s central role in organizing the RAF–MEK–ERK signaling module, targeting KSR1 alone may not fully suppress oncogenic signaling due to compensatory feedback loops and parallel pathway activation. Therefore, rational combination therapies that simultaneously inhibit KSR1 and other critical signaling nodes hold strong promise for achieving durable antitumor responses.

#### 8.3.1. Combination with MEK or ERK Inhibitors

Pharmacological inhibition of KSR1 could sensitize tumors to downstream blockade of MEK or ERK. Because KSR1 facilitates MEK phosphorylation and stabilizes active MEK–ERK complexes, its disruption may enhance the efficacy of MEK/ERK inhibitors by weakening pathway reactivation and reducing the emergence of resistant clones. Preclinical evidence indicates that genetic suppression of KSR1 potentiates the cytotoxic effects of MEK inhibitors, suggesting a synergistic interaction [165]. This approach could be particularly relevant in HCC and RAS-driven tumors, where persistent ERK activity underlies adaptive drug resistance.

#### 8.3.2. Combination with Receptor Tyrosine Kinase (RTK) Inhibitors

Upstream RTKs such as EGFR, FGFR, and c-Met are major activators of RAS signaling in HCC. RTK inhibitors (e.g., sorafenib, lenvatinib) often show transient efficacy due to compensatory RAS/MAPK reactivation [134,136,139]. Co-targeting KSR1 could suppress this rebound activation by disrupting scaffold-mediated pathway reassembly. Such vertical blockade of the RTK–RAS–KSR–MEK–ERK axis might enhance tumor control and delay resistance development. Moreover, inhibiting KSR1 could help overcome the escape mechanisms mediated by cross-talk between MAPK and PI3K/AKT signaling cascades.

#### 8.3.3. Combination with Immunotherapies

Emerging evidence links MAPK pathway activation to an immunosuppressive tumor microenvironment, partly through upregulation of PD-L1 and suppression of cytotoxic T-cell infiltration [166,167]. Inhibiting KSR1 might attenuate these immunosuppressive signals by reducing ERK-driven transcriptional outputs, thereby enhancing the efficacy of immune checkpoint inhibitors such as anti-PD-1/PD-L1 antibodies. The immunomodulatory potential warrants systematic evaluation of KSR1 blockade in combination with immunotherapy, particularly in HCC, where immune evasion remains a major therapeutic challenge.

In summary, KSR1 constitutes an emerging molecular vulnerability in human cancers. Its scaffold function positions it as a master regulator of RAS/MAPK oncogenic signaling, offering novel opportunities for targeted intervention even in patients lacking canonical *RAS* or *RAF* mutations. Advances in structure-based drug design, proteolysis-targeting technologies, and rational combination strategies hold considerable promise for translating KSR1 inhibition into clinically viable therapies—potentially reshaping treatment paradigms for patients with limited options.

## Figures and Tables

**Table 1:** Accessory and Scaffold Proteins for RAS/MAPK Signaling Pathway.

Class	Protein	Binding Partner	Functional Impact	Reference
Anchoring	CNK1 (CNKSR1)	RAF-1, Src, KSR	Upon RAS activation, promotes RAF relocalization/oligomerization → enhances RAF/MEK/ERK activation	[4,21]
	FLOT1/2 (Flotillins)	CRAF, MEK1/2, ERK1/2	Support EGFR clustering/activation; upon stimulation enhance CRAF/MEK/ERK signaling	[4,21]
	LAT, NTAL	Grb2, Gads, PLCγ1, Sos1	Nucleate membrane-proximal signalosomes	[4,21]
	PAQR10/11	H-, N-, KRAS4A, RasGRP1	Anchor RAS to the Golgi → spatial control of signaling	[27]
	SEF (IL17RD)	p-MEK, p-ERK	Binds p-MEK at Golgi; potentiates RAF/MEK/ERK signaling	[21]
Docking	DOK1/2	Activated RTKs (EGFR, RET) via PTB	Docking adaptors that assemble signaling complexes	[21]
	IRS2	Insulin receptor (RTKs)	Couples RTKs to downstream effectors	[21]
	β-arrestin1/2	GPCRs; RAF/MEK/ERK	Scaffolds RAF/MEK/ERK at plasma membrane and endosomes	[21]
	FRS2	RTKs, SHP2, GRB2	Central docking adaptor linking RTKs to Ras/ERK	[63]
Adapter	CRK/CRKL	Multiple pTyr ligands	Promotes ERK signaling by coupling pTyr scaffolds to C3G → Rap1 → B-Raf → MEK → ERK	[36]
	Grb2	pTyr proteins (SH2); SOS1 (N-SH3); Gab1 (C-SH3)	Core adaptor coupling RTKs to RAS via SOS	[37]
	Shc (ShcA/SHC1)	RTKs/integrins → Grb2	RTK-induced phosphorylation enables Grb2 binding to activate RAS	[40]
	SHP2 (PTPN11)	Gab1 (EGFR and other RTKs), FRS2 (FGFRs)	Tyr phosphorylation (e.g., Y542) relieves autoinhibition → supports RAS pathway	[41]
	Paxillin	ERK/MAPK module components	Recruits/organizes ERK module at adhesions	[42]
Scaffold	KSR1 and 2	RAF (BRAF, CRAF), MEK1/2, ERK1/2	Scaffold RAF–MEK–ERK at the membrane to boost efficient and precise RAS signaling	[64,65]
	IQGAP1	EGFR (direct/via ShcA), HER2 heterodimers	Bridges EGFR/HER2 to MAPK; tunes receptor trafficking and signal amplitude	[46]
	SHOC2	MRAS, PP1, RAF-1	Dephosphorylates inhibitory RAF-1 Ser259 site → enables RAF activation	[46,66,67,68]
Negative Modulator	SPRED family (SPRED1 and 2)	NF1	Negative regulation of RTK→RAS–MAPK by modulating RasGAPs	[49]
	Sprouty family (SPRY 1–4)	GRB2 (SH2)	Sequesters GRB2 away from SOS → suppresses RAS activation	[51]
	RKIP (PEBP1)	Raf-1, MEK	Blocks Raf-1/MEK interaction or binds Raf-1 N-region to inhibit MEK phosphorylation	[52]
	Erbin	ERBB2, Shoc2	Modulates Shoc2–ERK signal strength	[47]
	DUSP	ERK1/2	Acts as negative feedback regulators → directly dephosphorylates MAPKs (especially ERK)	[59,60]

**Table 2:** Targeting Components and Regulators of RAS/MAPK Signaling in HCC.

Agent	Target	Model	Result	Reference
rigosertib	RAS	in vitro (I)	Reduced HCC cell proliferation	[112]
miR-4510	RAF	in vitro (I)	Suppressed HCC cell proliferation	[113]
		in vivo (I)	Decreased tumor size and proliferation	
cobimetinib	MEK	in vitro (I)	Reduced HCC cell proliferation	[114]
		in vivo (I)	Decreased tumor development	
siRNA	ERK	in vitro (I)	Decreased HCC cell proliferation	[115]
RNF173	GRB2	in vitro (I)	Inhibited RAS/MAPK signaling	[117]
shRNA SUMO1	SHP2	in vitro (I) in vivo (A)	Inhibited RAS/MAPK signaling Increased tumor growth	[120]
miR-126 miR-126 inhibitor	SPRED1	in vitro (I) in vivo (A)	Increased proliferation and ERK phosphorylation Reduced tumor growth	[122]
SPRED2 siRNA	SPRED2	in vitro (A) in vivo (I)	Reduced proliferation and ERK phosphorylation Increased tumor growth	[123]
siRNA RKIP	RKIP	in vitro (I) in vitro (A)	Increased MEK phosphorylation Reduced HCC cell proliferation and migration	[126]

(I) Inhibition of target; (A) Activation of target.

**Table 3:** Resistance to Molecular Target Therapy in HCC.

Drug	Target	Resistance Mechanism	Reference
Lenvatinib	VEGFR, FGFR, PDGFR	EGFR–PAK2–ERK5 signaling activation	[134]
		Loss of NF1 and DUSP9	[135]
Sorafenib	VEGFR, FGFR, PDGFR, RAF	Overexpression of FGF	[136]
		Activation of HGF/c-Met axis	[139]
		Activation of EGFR and HER3	[140]
		Enrichment of cancer stem cells	[141]
		Compensatory activation of the PI3K/AKT pathway	[143]
Regorafenib	VEGFR, FGFR, PDGFR	Activation of TGF-β signaling	[150]
		NF-κB and STAT3 activation (SphK2 overexpression)	[151]

## Data Availability

Not applicable.
